# A novel patient-derived meningioma spheroid model as a tool to study and treat epithelial-to-mesenchymal transition (EMT) in meningiomas

**DOI:** 10.1186/s40478-023-01677-9

**Published:** 2023-12-15

**Authors:** Laurien L. van de Weijer, Emanuela Ercolano, Ting Zhang, Maryam Shah, Matthew C. Banton, Juri Na, Claire L. Adams, David Hilton, Kathreena M. Kurian, C. Oliver Hanemann

**Affiliations:** 1 Faculty of Health: Medicine, Dentistry and Human Sciences, Derriford Research Facility, University of Plymouth, Plymouth, PL6 8BU Devon UK; 2 Faculty of Health: School of Biomedical Sciences, University of Plymouth, Plymouth, PL4 8AA Devon UK; 3 Department of Cellular and Anatomical Pathology, University Hospitals Plymouth NHS Trust, Derriford, Plymouth, PL6 8DH Devon UK; 4 University of Bristol Medical School & North Bristol Trust, Southmead Hospital, Bristol, BS1 0NB UK

**Keywords:** Meningioma, Spheroids, EMT, MERTK, HDAC, Combination therapy

## Abstract

Meningiomas are the most common intracranial brain tumours. These tumours are heterogeneous and encompass a wide spectrum of clinical aggressivity. Treatment options are limited to surgery and radiotherapy and have a risk of post-operative morbidities and radiation neurotoxicity, reflecting the need for new therapies. Three-dimensional (3D) patient-derived cell culture models have been shown to closely recapitulate in vivo tumour biology, including microenvironmental interactions and have emerged as a robust tool for drug development. Here, we established a novel easy-to-use 3D patient-derived meningioma spheroid model using a scaffold-free approach. Patient-derived meningioma spheroids were characterised and compared to patient tissues and traditional monolayer cultures by histology, genomics, and transcriptomics studies. Patient-derived meningioma spheroids closely recapitulated morphological and molecular features of matched patient tissues, including patient histology, genomic alterations, and components of the immune microenvironment, such as a CD68 + and CD163 + positive macrophage cell population. Comprehensive transcriptomic profiling revealed an increase in epithelial-to-mesenchymal transition (EMT) in meningioma spheroids compared to traditional monolayer cultures, confirming this model as a tool to elucidate EMT in meningioma. Therefore, as proof of concept study, we developed a treatment strategy to target EMT in meningioma. We found that combination therapy using the MER tyrosine kinase (MERTK) inhibitor UNC2025 and the histone deacetylase (HDAC) inhibitor Trichostatin A (TSA) effectively decreased meningioma spheroid viability and proliferation. Furthermore, we demonstrated this combination therapy significantly increased the expression of the epithelial marker E-cadherin and had a repressive effect on WHO grade 2-derived spheroid invasion, which is suggestive of a partial reversal of EMT in meningioma spheroids.

**Supplementary Information:**

The online version contains supplementary material available at 10.1186/s40478-023-01677-9.

## Introduction

Meningiomas are the most common intracranial brain tumours and account for approximately 36% of all primary tumours of the central nervous system (CNS) [1]. The World Health Organisation (WHO) classifies meningiomas into WHO grade 1 (benign), WHO grade 2 (atypical), and WHO grade 3 (anaplastic) [2]. WHO grade 1 meningiomas (80%) have a good prognosis with an estimated 10-year overall survival of 80–90%, while WHO grade 2 (15–18%) and grade 3 (2–4%) are more aggressive and have a high risk of recurrency [3, 4]. Indeed, 10-year overall survival for high grade 3 meningiomas is estimated as 14–34% [4]. Most meningiomas, particularly benign grade 1 tumours, can be successfully treated by surgical resection and/or radiotherapy [5]. However, these therapies have been associated with postoperative morbidities and radiation neurotoxicity [5]. Currently, there is a lack of systemic treatment for meningiomas. Over the last decade, the availability of advanced sequencing technology (‘next-generation sequencing') has resulted in a comprehensive understanding of the genetic background of meningiomas and the mutations (NF2, TRAF7, AKT1, KLF4, SMO, POLR2A, PIK3CA, SMARCE1, SMARCB1, hTERT, CDKN2A/2B) underlying tumour development and progression [6–10]. Furthermore, novel molecular classifications that more reliably reflect tumour behaviour compared to the WHO grading system have been suggested [11]. Despite the advances that have been made in the understanding of the genetic background and mutational landscape of meningiomas, progress in the development of therapeutic approaches targeting genetically stratified tumours remains limited [1, 6, 12]. Hence, the development of effective drug-based therapeutics is imperative.

The diverse immune microenvironment of meningiomas has been demonstrated to influence meningioma pathogenesis [13, 14]. For example, a high degree of macrophage infiltration (CD68 + macrophages and CD68 + CD163 + M2 macrophages) has been associated with tumour aggressiveness and therapy resistance of meningiomas. In addition, immunotherapy for the treatment of meningiomas, including immune checkpoint inhibitors, are currently under investigation [15–18]. Therefore, the use of experimental models that can closely resemble patient tumours, including the immune microenvironment, and thus predict therapy response, is crucial to investigate effective molecular therapies for meningiomas [19–21]. A well-established method that is often used to test the therapeutic response to novel drug compounds is the use of patient-derived cells. These cells are typically propagated as two-dimensional (2D) monolayers [22]. However, 2D monolayer cultures have limited predictive value due to the highly artificial culture conditions of being attached to the flat surface of culture dishes [22, 23]. Therefore, more complex three-dimensional (3D) cell culture methods have been developed and demonstrated as more relevant in vitro experimental tools for tumour modelling over conventional 2D monolayer culture [23]. Specifically, 3D cultures harbour the power to resemble the in vivo tumour with respect to tissue-specific architecture, cell–cell and cell-microenvironment interactions, growth patterns and penetration gradients of oxygen and drugs [23]. Hence, development of 3D model systems for meningiomas can improve the accuracy of drug developmental studies by modelling patient-specific characteristics and the immune microenvironment [24]. In this study, we developed a novel easy-to-use patient-derived meningioma spheroid model as a meningioma drug development tool. Extensive characterisation demonstrated that this novel spheroid model recapitulates important histological and molecular features of patient tissues such as the maintenance of diverse cell populations, including tumour cells and macrophage populations and the maintenance of genetic alterations.

Epithelial-to-mesenchymal transition (EMT) is a common oncogenic process associated with therapeutic tumour progression, treatment resistance, invasion capacity and poor prognosis [25–28]. It describes the process of epithelial cells that lose expression of their typical epithelial proteins (e.g. E-cadherin), while acquiring expression of mesenchymal proteins (e.g. N-cadherin, vimentin, fibronectin). These changes are orchestrated by several EMT-associated transcription factors including Slug, Snail, and Zeb1, that regulate E-cadherin expression [28]. In meningiomas, low E-cadherin and high Slug expression have been correlated with recurrent tumours, suggesting the involvement of EMT in meningioma progression [28]. Therefore, this oncogenic process is an interesting target for the treatment of meningiomas. Comprehensive transcriptomic analysis comparing our newly established spheroid model with traditional monolayer cultures revealed the upregulation of genes associated with EMT and the Notch signalling pathway, demonstrating the suitability of this novel spheroid model to study EMT. Using our newly established spheroid model, we provided evidence for the therapeutic potential of inhibition of MER tyrosine kinase (MERTK), a receptor tyrosine kinase (RTK) that has previously been described to contribute to EMT [29], in combination with HDAC inhibition for the treatment of WHO grade 1 and grade 2 meningiomas. We demonstrated that this combination strategy using the FTL/MERTK inhibitor UNC2025 and Histone deacetylase (HDAC) inhibitor Trichostatin A (TSA) synergistically decreased spheroid viability, and decreased spheroid proliferation [30, 31]. Furthermore, the results presented here indicated that combination therapy of UNC2025 and TSA can decrease the mesenchymal phenotype and spheroid invasion capacity.

## Materials and methods

### Human tumour specimens

Meningioma specimens were obtained with written informed consent of all participating patients after the national ethical approvals (Plymouth Brain Tumour Biobank, South Central—Hampshire B Research Ethics Committee, REC No: 19/SC/0267, IRAS project ID: 246,667). All samples were de-identified prior to processing and given a unique identification number (‘MN’). Clinical and histopathological data for all samples used in this study are listed in Table 1. All meningioma tumours were graded by a neuropathologist. Specimens were collected during surgery and immediately placed in Hibernate™ A medium (Thermo Fisher Scientific) supplemented with transport medium containing 1% Amphothericin B (Merck) and 100 U/mL penicillin/streptomycin (Thermo Fisher Scientific).

**Table 1:** Clinical and histopathological data for patient samples

Patient	Histopathological subtype	Grade	Age	Gender (F = female, M = male)
MN504	Unknown	1	44	F
MN490	Mixed meningothelial & microcystic	1	Unknown	Unknown
MN523	Meningothelial	1	79	M
MN525	Fibrocollagenous	1	75	M
MN595	Fibroblastic	1	56	F
MN609	Transitional	1	62	F
MN611	Transitional	1	78	F
MN613	Transitional	1	64	F
MN614	Transitional	1	46	T
MN656	Psammomatous	1	78	F
MN655	Secretory	1	65	F
MN658	Transitional	1	58	F
MN485	Meningothelial	1	Unknown	Unknown
MN486	Meningothelial	1	Unknown	Unknown
MN487	Fibrous	1	Unknown	Unknown
MN493	Fibrous	1	Unknown	Unknown
MN554	Transitional	1	Unknown	Unknown
MN577	Psammomatous	1	75	Unknown
MN557	Unknown	1	43	F
MN567	Meningothelial	1	61	F
MN592	Meningothelial	1	Unknown	Unknown
MN602	Transitional	1	58	M
MN630	Atypical	2	58	M
MN566	Psammomatous	1	62	F
MN635	Meningothelial	1	66	F
MN610	Secretory	1	30	F
MN588	Meningothelial	1	Unknown	Unknown
MN429	Meningothelial	1	70	F
MN460	Meningothelial	1	54	F
MN461	Meningothelial	1	Unknown	Unknown
MN472	Transitional	1	56	F
MN474	Psammomatous	1	65	F
MN467	Meningothelial	1	45	F
MN408	Transitional	1	25	F
MN414	Psammomatous	1	45	F
MN437	Meningothelial	1	Unknown	F
MN481	Fibrous	1	Unknown	F
MN553	Transitional	1	Unknown	M
MN581	Meningothelial	1	34	F
MN233	Transitional	1	37	F
MN231	Transitional	1	58	F
MN329	Fibrous	1	Unknown	Unknown
MN313	Meningothelial	1	Unknown	Unknown
MN498	Angiomatous/microcystic	1	48	M
MN465	Transitional	1	75	M
MN440	Atypical	2	Unknown	Unknown
MN521	Atypical	2	62	F
MN409	Atypical	2	Unknown	Unknown
MN428	Atypical	2	42	F
MN603	Atypical	2	67	M
MN605	Atypical	2	64	F
MN660	Atypical	2	Unknown	Unknown
MN582	Atypical	2	64	F

Histopathological subtype, WHO grade, age and gender (M = male, F = female) for each patient (MN)

### Tissue processing

Specimens were collected during surgery and immediately placed in transport medium. Samples were washed twice in sterile 1X Phosphate Buffered Saline (PBS) (Thermo Fisher Scientific) and transferred to a 100 mm culture dish containing complete meningioma WHO grade 1 medium (MN1: Dulbecco’s Modified Eagle Medium (DMEM) (Thermo Fisher Scientific), 10% (v/v) FBS (Merck), 100 U/mL penicillin/streptomycin, 1% (v/v) GlutaMAX™-I (Thermo Fisher Scientific) or complete meningioma WHO grade 2 medium (MN2: DMEM/ F12 Nutrient Mixture (Ham) (1:1) (Thermo Fisher Scientific), 20% (v/v) FBS (Merck), 100 U/mL penicillin/streptomycin (Thermo Fisher Scientific), 1% (v/v) GlutaMAX™-I (Thermo Fisher Scientific) in a laminar flow cabinet. Tissue was dissected using a sterile scalpel (VWR International Ltd, 0507 n.21). Areas with substantial necrosis were removed and tumour pieces were snap frozen and saved for extraction of DNA, RNA (2 mm^2^) and protein (5mm^2^). Resected tumours were further dispersed into single cells using sterile curved dissection scissors (VWR International Ltd, Z265977) and by pipetting up and down several times using a 10 ml sterile plastic pipette. The cell suspension was collected in a canonical 50 ml tube and incubated in 1X Red Blood Cell (RBC) lysis buffer (Thermo Fisher Scientific, eBioscience™) for 10 min under gentle rotation. Cells were pelleted, washed in 1X PBS (Thermo Fisher Scientific), and resuspended in complete meningioma medium. Cell suspensions were strained using a cell strainer with a 100 mm nylon mesh (Thermo Fisher Scientific) to remove cellular debris and seeded into several 25-cm2 cell culture flasks (Greiner Bio-One) according to tumour size or cryopreserved. Cell culture flasks were placed into an incubator at 37 °C in a humidified atmosphere (5% CO_2_). Cell medium was replaced every 3 days.

### Spheroid culture

For spheroid culture, primary cells from the tumour at passage 0 (P0) were detached using 0.25% Trypsin/EDTA (Thermo Fisher Scientific), washed in 1X PBS (Thermo Fisher Scientific) and resuspended in complete spheroid growth medium (GFS) (DMEM/Nutrient Mixture F12 and Neurobasal (Thermo Fisher Scientific) at a 1:1 ratio, 5% (v/v) FBS (Merck), 1X B27-supplement (Thermo Fisher Scientific), 1X N2-supplement (Thermo Fisher Scientific), 20 ng/ml recombinant human epidermal growth factor (EGF) protein (Bio-Techne), 20 ng/ml recombinant human basic fibroblast growth factor (bFGF) protein (Bio-Techne), 100 U/mL penicillin/streptomycin (Thermo Fisher Scientific), 1% (v/v) GlutaMAX™-I (Thermo Fisher Scientific), 1% (v/v) non-essential amino acids (NEAA) (Thermo Fisher Scientific). Cells were counted and seeded at 3000 cells/well in U-shaped ultra-low adherend (ULA) 96-well microplates (Greiner Bio-One, 650979). Culture plates were centrifuged at 1500 rpm for 15 min and placed in a were placed into an incubator at 37 °C in a humidified atmosphere (5% CO_2_) under constant rotation (65 rpm). Spheroids were left in the incubator for 3 days to allow spheroid formation. Spheroids were exclusively formed from cells attached at passage P0, forming passage P1 spheroids.

### Spheroid growth analysis

To measure the growth of spheroids, images of individual spheroids were routinely obtained using brightfield microscopy. The maximal (max.) diameter was measured by using the measuring tool on ImageJ. The max. diameter at day 3 was taken to calculate the growth ratio for each following time point. The ratios were plotted on a growth curve visualising the growth. Volume was calculated using the formula V = π · ø^3^/6, with V = spheroid volume, ø = diameter.

### Immunohistochemistry

Spheroids were fixed six days post seeding, which corresponds to three days post formation, and tissues were fixed immediately after resection. Tissues and spheroids were fixed in 16% (v/v) formaldehyde followed by dehydration, paraffin embedding and sectioning. Paraffin Sects. (4 µm) were de-waxed, rehydrated and stained with Hematoxylin and Eosin (H&E) staining. For immunodetection, sections were stained using the primary antibodies including CD68 (1:50) (Agilent Cat# M0876, RRID:AB_2074844), CD163 (1:50) (Roche Cat# 05973929001, RRID:AB_2335969), E-cadherin (1:50) (Agilent Cat# M3612, RRID:AB_2076672), SSTR2 (1:400) (Abcam Cat# ab134152, RRID:AB_2737601), Vimentin (1:2000) (Agilent Cat# M0725, RRID:AB_10013485), Ki67 (1:100) (Agilent Cat# M7240, RRID:AB_2142367) using the Ventanna automated machine. Nuclei were counterstained with haematoxylin (Merck). For spheroid analysis, a minimum of 3 spheroids were analysed per sample. For tissue analysis, 3 independent fields were counted. For each field, > 1000 cells were counted.

### Genomic analysis

Total genomic DNA was extracted from frozen meningioma tissues and matched spheroids seeded at P1 (3 days post spheroid formation), using the DNeasy® Blood and Tissue kit (QIAgen, 69,504) following manufacturers’ instructions. DNA concentrations and quality was estimated using the NanoDrop Spectrophotometer. DNA was sequenced by the South West Genomic Laboratory Hub using the Illumina TruSight Oncology 500 panel. The raw sequence data was analysed using the TruSight Oncology 500 v2.2 Local App. Next, variant calling data was processed using the online servers Cancer Genome Interpreter (RRID:SCR_023752) [32, 33] and wANNOVAR (RRID:SCR_000565) [34–36] to identify and annotate the driver mutations. All driver mutations were filtered based on variant sample coverage (≥ 90% at 50X according to the set threshold by the TSU500 local app), allele frequency (VF, ≥ 0.05) [37], read depth (DP ≥ 100) [38, 39], ExAC (≤ 0.05) [40] and fathmm_MKL score prediction (D = damaging) [41, 42]. All filtered drivers between spheroids and parent tumours were compared to identify the common variants.

### RNA isolation and gene expression analysis

Total RNA was extracted from patient matched cell monolayers, spheroids and meningioma tissues using the Direct-zol™ RNA MiniPrep kit (Zymo Research) following manufacturers' instructions. Quantification and quality was carried out using the NanoDrop Spectrophotometer.

RT-PCR was performed using 500 ng of total RNA with the High-Capacity cDNA Reverse Transcription Kit (Thermo Fisher Scientific). Real Time PCR (qPCR) was performed using the TaqMan® Fast Advanced Master Mix supplemented with TaqMan® assays (Thermo Fisher Scientific) on a LightCycler® 480 II System (Roche), in three technical triplicates using the following probes: CDH1 (Hs01023895_m1), GAPDH (Hs02786624_g1), Hes1 (Hs00172878_m1), Hey1 (Hs01114113_m1), Notch1 (Hs01062014_m1), RPL37A (Hs01102345_m1), Snail1 (Hs00195591_m1), Snail2 (Hs00161904_m1), Zeb1 (Hs00232783_m1), ZO1 (Hs01551861_m1). Gene expression levels were calculated using the quantitative 2 − (ΔΔCt) method [43].

### Messenger RNA (mRNA) sequencing and data analysis

For transcriptomic analysis, isolated RNA was sent to Novogene where RNA integrity was assessed and assigned an RNA Integrity Number (RIN). Samples with RIN > 7 were processed for sequencing. mRNA was purified from total RNA using poly-T oligo-attached magnetic beads and cDNA libraries were generated. Libraries were quantified using Qubit. Libraries were sequenced using the NovaSeq 6000 PE150 Illumina platform and paired-end reads were generated. Raw data (raw reads) of fastq format were processed to generate clean data (clean reads). Reads containing adapters, reads containing poly-N and low-quality reads were removed from raw data. Q20, Q30 and GC content of clean data were calculated. All downstream analyses were based on clean data with high quality. Paired-end clean reads were aligned to the human reference genome homo_sapiens_ensemble_94 using Hisat2 v2.0.5 (RRID:SCR_015530). The tool ‘featureCounts v1.5.0-p3’ (RRID:SCR_012919) was used to count the reads numbers mapped to each gene. Fragments per Kilobase of transcripts per Million mapped reads (FPKM) of each gene was calculated based on the length of the gene and the reads count mapped to this gene. Differential expression analysis was performed using the DESeq2 Rpackage (1.20.0) (RRID:SCR_015687). Resulting P-values were adjusted using the Benjamini and Hochberg’s approach for controlling the false discovery rate (FDR). Genes with an adjusted *P*-value < 0.05 were assigned as differentially expressed. For Gene Set Enrichment Analysis (GSEA), genes were ranked according to the degree of differential expression and the predefined gene sets (GO) were tested for enrichment. The local version of the GSEA analysis tool https://www.gsea-msigdb.org/gsea/index.jsp was used.

### Western blotting

Cells and spheroids were lysed in radioimmunoprecipitation assay lysis buffer (RIPA) (Cat# 89900, Thermo Fisher Scientific) (approximately 50 µL per 96 spheroids) containing Halt™ Protease and Phosphatase Inhibitor cocktail (Thermo Fisher Scientific). Spheroids were subjected to 3 cycles of freezing in liquid nitrogen and thawing in a heat block at 37 °C. Spheroids were sonicated for 2 cycles of 2 min sonication and 1 min rest on ice, using a water bath sonicator (Grant Ultrasonic bath XUBA1) to ensure complete spheroid lysis, centrifuged at maximum speed for 15 min and stored at − 80 °C. Western blotting was performed as described previously [44]. Membranes were incubated with primary antibodies against E-cadherin (1:500) (Cell Signaling Technology Cat# 3195, RRID:AB_2291471), Hey1 (1:1000) (Abcam Cat# ab154077, RRID:AB_2893447), Hes1 (1:1000) (Cell Signaling Technology Cat# 11,988, RRID:AB_2728766), Notch1 (1:1000) (Cell Signaling Technology Cat# 3608, RRID:AB_2153354), Slug (1:1000) (Cell Signaling Technology Cat# 9585, RRID:AB_2239535), Snail (1:500) (Cell Signaling Technology Cat# 3879, RRID:AB_2255011), N-cadherin (1:500) (Cell Signaling Technology Cat# 13,116, RRID:AB_2687616) and GAPDH (1:10,000) (Millipore Cat# MAB374, RRID:AB_2107445). Horseradish peroxidase-conjugated secondary antibodies (1:5000) (Bio-Rad Cat# 170–6515, RRID:AB_11125142, Cat# 1,706,516, RRID:AB_2921252) and chemiluminescence (Thermo Fisher Scientific) were used for the detection of immunoreactive bands. ImageJ (RRID:SCR_003070) software was used for densitometry quantification of protein bands.

### Drug treatment and dose–response analysis

Spheroids and monolayer cultures were treated with the following inhibitors: MERTK/Flt3 inhibitor (UNC2025) (CAS 2070015–17-5) (Cambridge Bioscience, CAY166130), HDAC inhibitor Trichostatin A (TSA) (CAS 58880–19-6) (Stratech, S1045-SEL). Monolayer cultures were seeded at 3000 cells/well in a volume of 100µL in opaque-walled flat-bottom 96-well plates (Corning™) 24 h prior to treatment. Growth medium was replaced with fresh complete medium containing the drug at desired concentrations. Control wells were treated with empty vehicle (DMSO or ethanol) at a maximum concentration of 0.001% (v/v). Spheroids were seeded at 3000 cells/100µL per well in U-shaped ULA 96-well microplates (Greiner Bio-One, 650979) 3 days prior to treatment. Following spheroid formation, 50µL of media was carefully aspirated from each well without disturbing the spheroid and replaced with 50 µL of fresh GFS containing the desired concentration of each inhibitor. Wells were incubated with drug for 72 h at 37 °C and 5% CO_2_. Cell viability was detected using the CellTiter-Glo 2.0 Cell viability assay (Promega, G9242) for monolayers or CellTiter-Glo 3D Cell Viability Assay (Promega, G9682) for spheroids according to manufacturer’s recommended protocol. Total cell numbers were determined as a percentage of vehicle (EtOH or DMSO). In experiments where direct comparisons were made between culture methods, patient and passage-matched samples were used and seeded simultaneously.

### 3D invasion assay

Media was carefully aspirated without disturbing the spheroid 72 h post-seeding. Matrigel™ Basement Membrane Matrix (Thermo Fisher Scientific, 356234) was thawed on ice and 80 µL was carefully added to each well to embed spheroids in Matrigel™ drops avoiding air bubbles. Spheroids were gently positioned in the centre of the well using a pipet tip. Microplates were placed in the incubator for 30 min to allow Matrigel™ to set. When Matrigel was solidified, 100 µL of GFS was added to each well. In drug experiments, the drug was added to the media at desired concentrations. Spheroids were assessed for cell invasion 24 and 48 h post-embedding using bright-field microscopy at 10 × magnification (Leica, IM8). Invasion was measured as max. diameter of invaded area using ImageJ (RRID:SCR_003070) software.

### Immunofluorescence

Immunofluorescence staining was performed on spheroids following 48 h drug treatment. Spheroids were fixed in 4% (v/v) paraformaldehyde (Thermo Fisher Scientific) for 30 min. Spheroids were washed in 1X PBS (Thermo Fisher Scientific) and permeabilized using 0.5% (w/v) Triton-X-100 (Sigma Aldrich) for 1 h at room temperature. Non-specific binding of antibodies was blocked by incubating spheroids in blocking buffer containing 1% (w/v) Bovine Serum Albumin (Fisher Scientific) and 10% (v/v) normal goat serum (Abcam) in 1X PBS for 2 h at room temperature. Spheroids were incubated with primary antibody: anti-Ki67 (MIB-1) (1:100) overnight at 4 °C (Agilent Cat# M7240, RRID:AB_2142367). Secondary antibody goat anti-mouse IgG Alexa Fluor 594 (Thermo Fisher Scientific Cat# A-11005, RRID:AB_2534073) (1:250) was used to visualize primary antibody. DAPI (1:500) (cat# D9542, Thermo Fisher Scientific) was used for nuclear counterstaining. Fluorescence images were taken using 40 × objectives on Leica SP8. Images were processed with ImageJ (RRID:SCR_003070) software.

### Statistical analysis

Statistical analysis was performed using the paired Student’s t-test in experiments with two groups, and one-way ANOVA in experiments with three or more groups with Tukey’s multiple comparison test as post-hoc analysis using GraphPad prism software, except when indicated otherwise in figure legend. Repeats of experiments were performed with different patient samples. Data are expressed as mean ± SEM. IC_50_ values were calculated using GraphPad Prism analysis software (RRID:SCR_002798).

## Results

### Development of a patient-derived meningioma spheroid model

**Fig.1:**
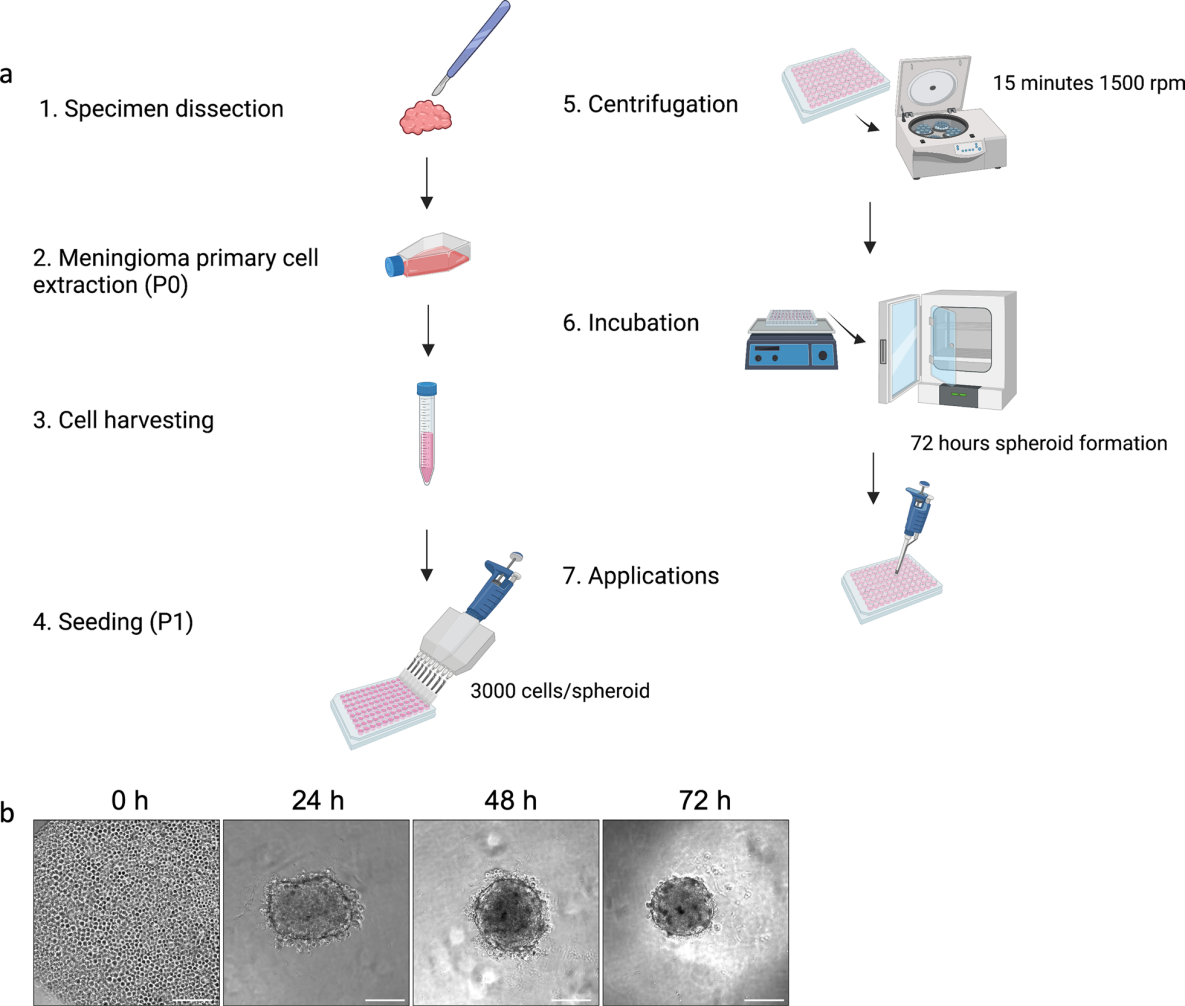
Establishment of 3D patient-derived meningioma spheroid model. **a** Schematic diagram of the protocol for the generation of patient-derived spheroids. **b** Representative brightfield images of spheroid formation: 0 h after centrifugation, 24 h cell aggregation, 48–72 h compact spheroid, ready for use in downstream applications. Scale bar: 200 µm. Figure was created using Biorender.com

We formed spheroids by seeding cells in 96-well u-bottom ultra-low adherence (ULA) plates at P1 with a success rate of 96% (Fig. 1a). To ensure generation of uniform-sized spheroids, culture plates were centrifuged after seeding to help spheroid compaction. Within 24 h, we observed formation of non-compact cellular aggregates which established the characteristic 3D structure at 2–3 days post-seeding, confirmed by compact round-shaped spheroids with a dense core and smooth edges (Fig. 1b). Clinical and histopathological information of tumours are listed in Table 1.

**Fig. 2:**
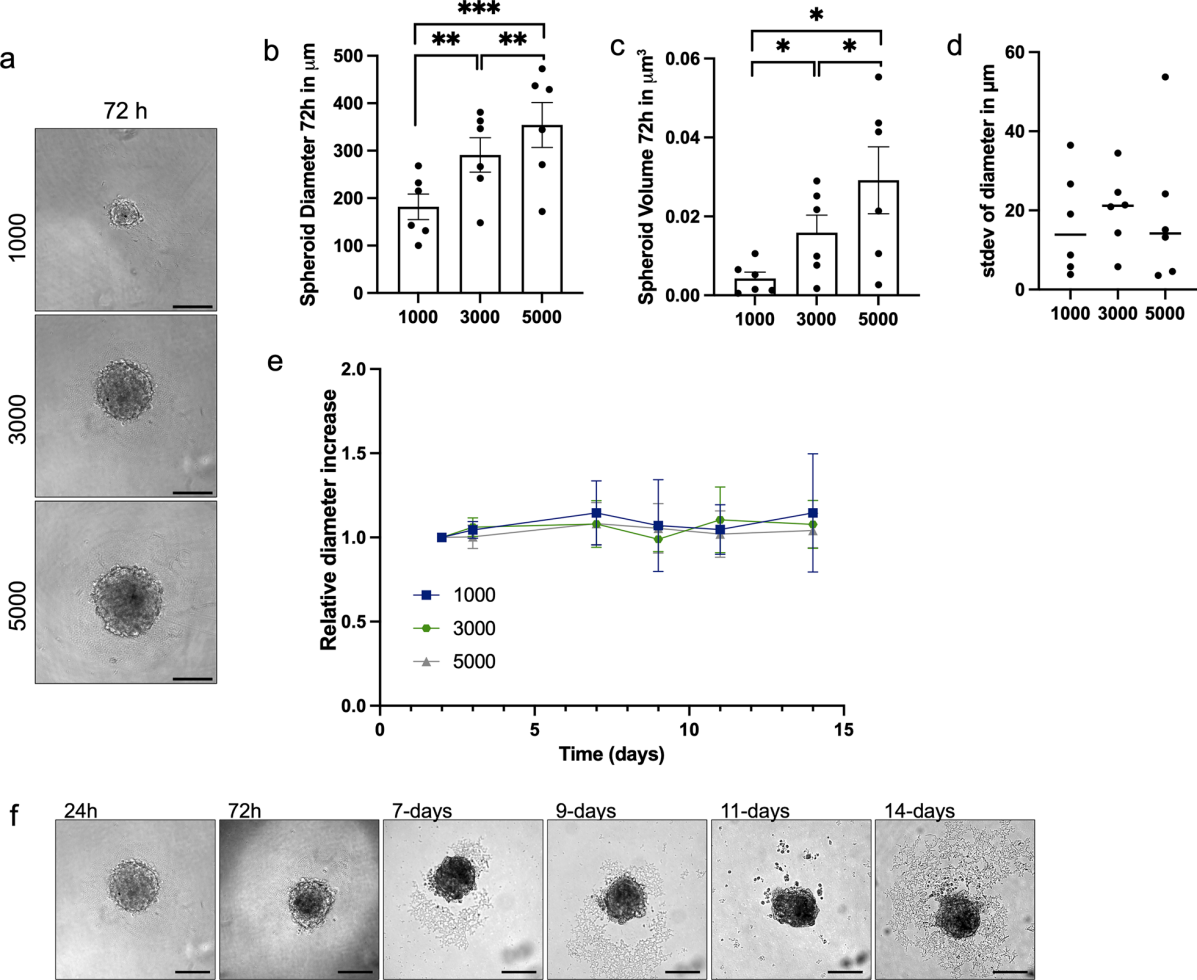
Patient-derived meningioma spheroid size is controlled by seeding density and remains stable over time. **a** Representative brightfield images of patient-derived meningioma spheroids 72 h post-seeding for seeding density of 1000, 3000 and 5000 cells. Scale bar in each panel: 200 µm (Leica IM8). **b**, **c** Bar graphs of (**b**) spheroid diameter in µm and (**c**) spheroid volume in µm^3^ 72 h post-seeding for spheroids of 1000, 3000 and 5000 cells (n = 6). **d** Dot plot showing the average standard deviations of spheroid diameter (µm) of spheroids generated from the same sample. **e** Relative fold change in spheroid diameter from spheroid formation to 14 days post-seeding. Each line represents the mean of 5 independent experiments ± standard error for 1000 cells (blue), 3000 cells (green) and 5000 cells (grey). **f** Representative bright-field images of spheroids over 14 days. Scale bar in each panel: 200 µm (Leica IM8). Paired t-test was used for statistical evaluation. *p < 0.05, **p < 0.01, ***p < 0.001

Next, we assessed spheroid spatial features and growth using brightfield microscopy (Fig. 2a). Spatial features were assessed directly after spheroid formation (72 h). The mean diameter at 72 h for spheroids seeded at a density of 1000, 3000 and 5000 cells was respectively 181.7 ± 66.1, 291.1 ± 89.1, 354.2 ± 115.6 µm (Fig. 2b). This corresponded to a mean volume of 4.27 ± 3.90, 15.85 ± 10.93, and 29.17 ± 20.57 µm^3^ (Fig. 2c). These values significantly increased with seeding density, demonstrating that spheroid size strongly depends on seeding concentration. Furthermore, the average standard deviations of spheroid diameter in µm of spheroids generated from the same sample were 16.8 ± 13.0, 20.2 ± 9.6 and 19.1 ± 18.5 for a seeding density of 1000, 3000 and 5000 cells, respectively, indicating the generation of uniform spheroids for each sample, despite the variability in spheroid diameter between samples (Fig. 2d). Spheroid diameter remained stable for 14 days for spheroids seeded at each density (Fig. 2e, f). Spheroids were also successfully generated from primary cells at higher passage numbers, but these were not further analysed (Additional file 4: Fig. S1).

### Meningioma spheroids exhibit histological and molecular features of parental tumour and resembles the tumour microenvironment

**Fig. 3:**
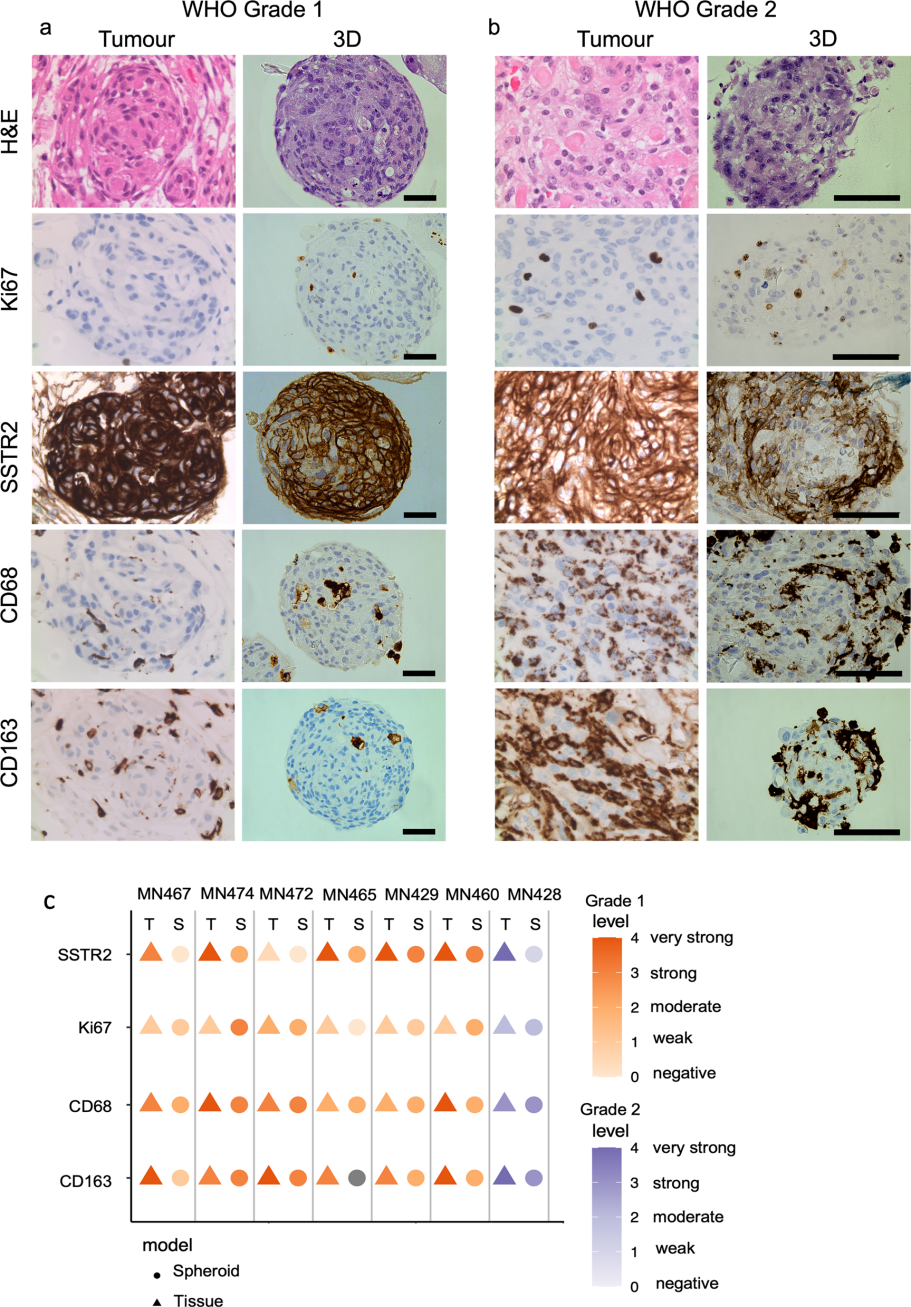
Patient-derived spheroids preserve histology, protein expression and immune components of matched tumour tissues. Representative immunostaining images of **a** WHO grade 1 (n = 6) and **b** WHO grade 2 (n = 1) of patient tumour tissues (Tumour) and matched patient-derived meningioma spheroids (3D). Spheroids were fixed and embedded six days after seeding, corresponding to three days after spheroid formation. Stainings are shown in order: H&E, anti-SSTR2, anti-Ki67, anti-CD68 and anti-CD163. Scale bars: 200 µm. **c** Plot of immunoscores of stainings displayed in a and b in tissues, T, (triangle) and spheroids, S, (circle) (n = 7). Colour represents immunoscores of 0–4 (0 = negative, 1 = weak, 2 = moderate, 3 = strong, 4 = very strong) in orange for grade 1 and purple for grade 2. Grey dot indicates no scoring

To investigate whether patient-derived spheroids resemble the in vivo tumour characteristics of matched parent tissues, we performed immunohistochemical analysis. Hematoxylin & Eosin (H&E) staining revealed that spheroids retained a solid structure resembling the anatomy of meningioma in vivo (Fig. 3a, b). Spheroids were observed to retain histological characteristics such as prominent nucleoli and showed comparable levels of cellularity. In addition, spheroids generated from WHO grade 1 and 2 tumours showed a similar immunoscore of the proliferation marker Ki67 as compared to matched patient tissues (Fig. 3a, b) and retained expression of somatostatin receptor 2 (SSTR2), a marker commonly expressed by meningioma cells, although this staining was weaker in spheroids compared to tissue (average immuno score of 1.6 in spheroids compared to average score of 3.3 in tissue) (Fig. 3c). However, the percentage of positive Ki67 stained cells was higher in spheroids compared to tissue. To confirm the presence of infiltrating macrophages in our spheroid model, we performed immunostaining analysis for the pan-macrophage marker CD68 and the M2 macrophage marker CD163 (Fig. 3a, b). We identified the presence of CD68 + and CD163 + macrophages in all spheroids, indicating that important microenvironmental interactions are maintained in WHO grade 1 (Fig. 3a) and WHO grade 2 (Fig. 3b) derived spheroids.

Simplycounting % of immune cells in parental tissue and spheroids numbers were similar, counting KI67 positive cells was higher in the spheroids.

**Fig. 4:**
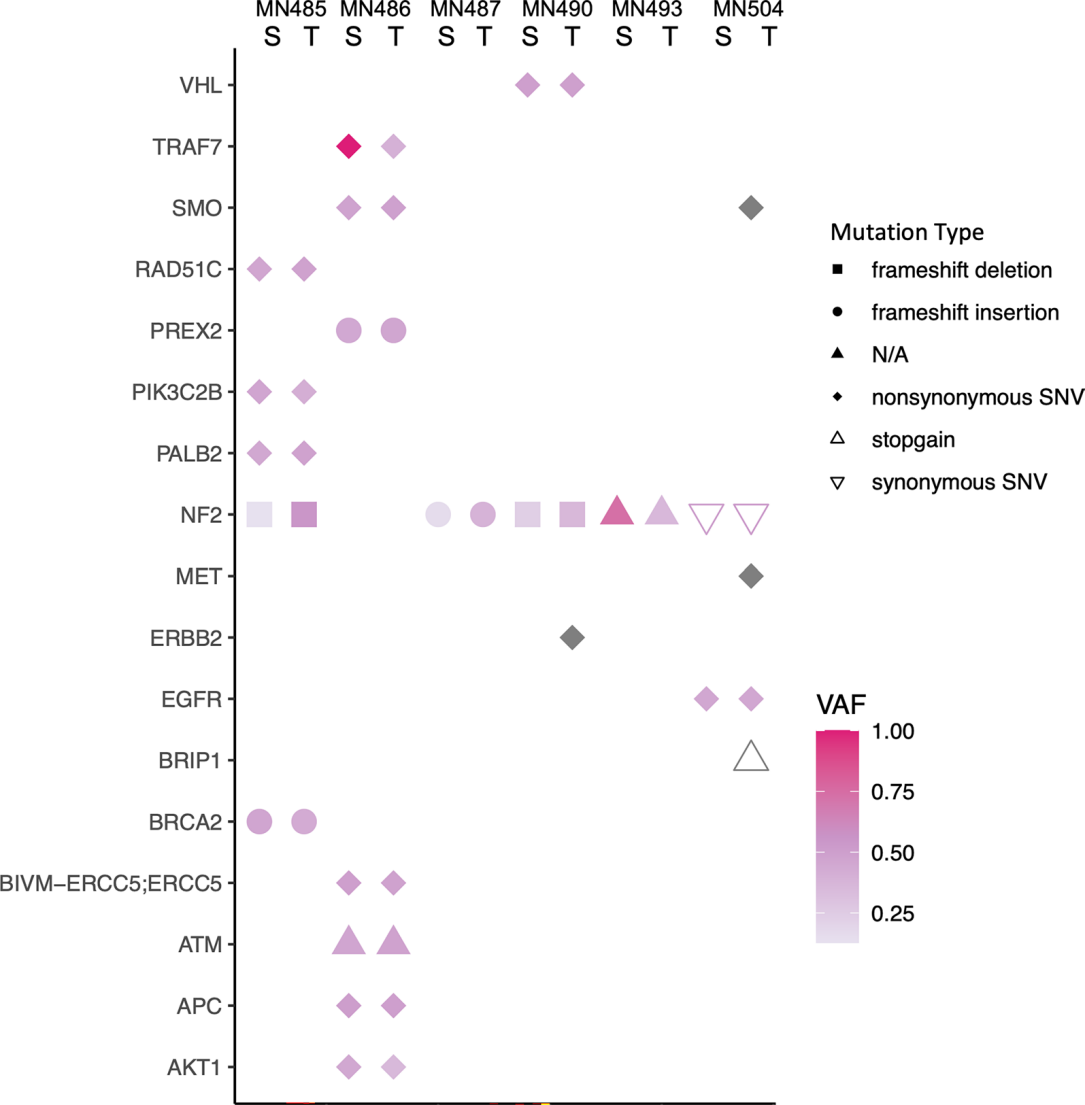
Patient-derived meningioma spheroids reflect the mutational profile of patient matched tumour tissues. Comparison of driver mutations detected in spheroids (S) and patient matched tumour tissues (T) (n = 6). Shapes indicate mutation type and colour represents variant allele frequency (VAF) as indicated on the right of the graph. Pink shapes are detected in spheroids and tissues, while grey shapes are only detected in tissues

Meningioma spheroids were further characterised to see whether they maintained the genomic alterations of their matched parent tissues. Genomic analysis revealed that, on average, 84.4% of all the identified driver mutations were preserved in the patient-derived spheroids, with 100% preservation in 4 out of 6 cases. Furthermore, we did not detect any novel driver mutations in the spheroids that could not be detected in the matched patient tissues. Important driver mutations specifically associated with meningioma pathology (including NF2, TRAF7, SMO, PIK3C2B and AKT1) that were identified in the tissues were consistently found in spheroids (Fig. 4). Details of all driver mutations identified in spheroids and tumour tissues can be found in Additional file 1. Additionally, comparison of the variant allele frequency (VAF) of detected driver mutations between spheroids and matched patient tumours revealed comparable frequency between both conditions (Fig. 4). This indicated that spheroid culture does not introduce genomic changes. Overall, these results showed that patient-derived meningioma spheroids conserved important histological and molecular features of parental tumours.

### Comparative transcriptomic analysis of meningioma spheroids with matched monolayers and parental tumour tissues

**Fig. 5:**
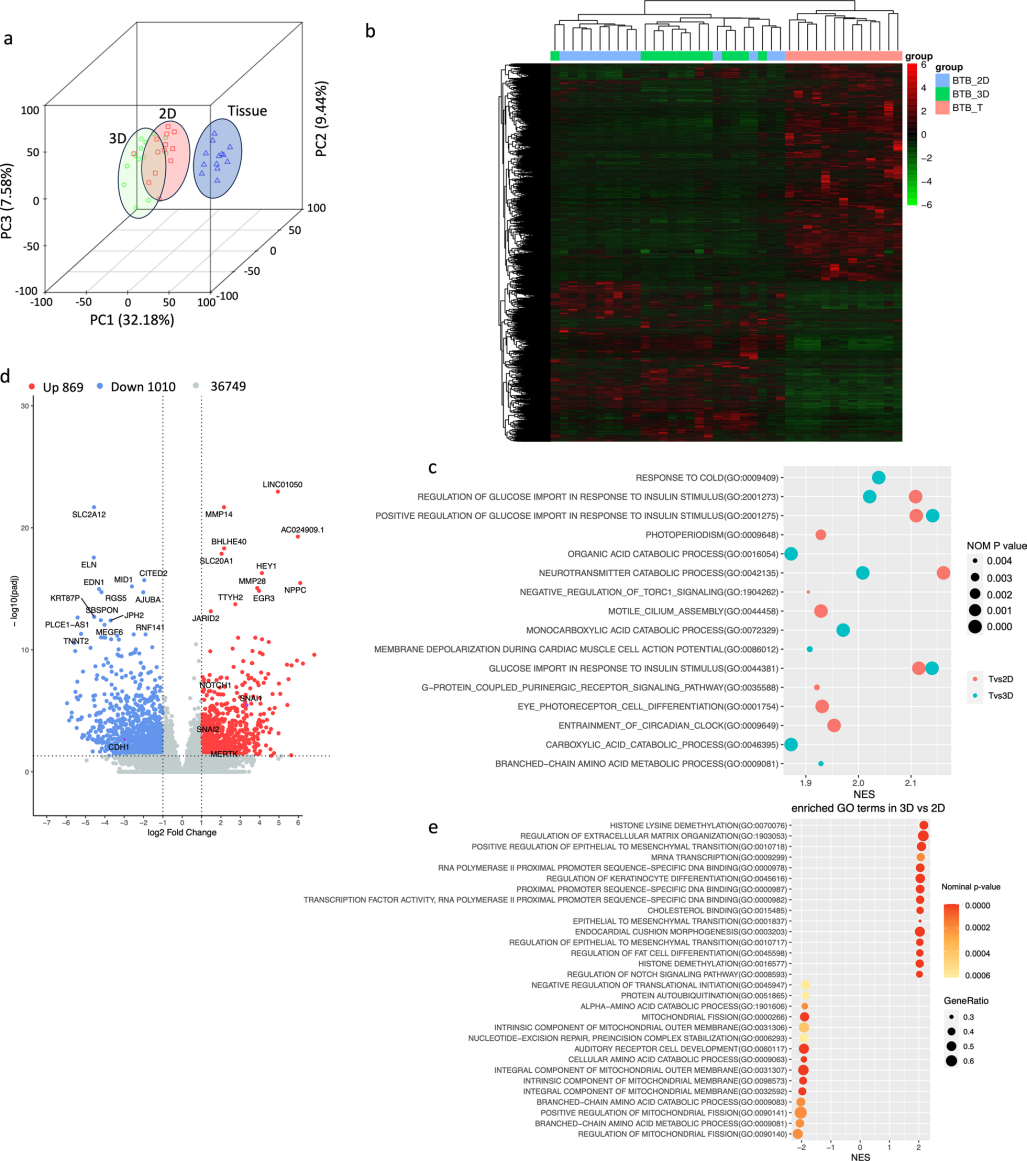
Patient-derived spheroid show distinct transcriptomic changes compared to monolayer cultures. **a** 3D Principal component analysis plot showing the variance between transcriptomes of patient tissues (blue triangles), 2D cultures (red squares), and 3D cultures (green circles) of matched patient material. **b** Heatmap of hierarchical clustering analysis of the logarithmic transformation of gene expression values. Samples are clustered into tissue (red) and cell culture, which splits into a 3D (green) and 2D (blue) cluster. **c** Scatter plot of GSEA analysis showing the top 10 most enriched gene ontology (GO) biological processes in tissues compared to 2D cultures (red) and 3D cultures (blue). Nominal enrichment score (NES) is represented on the x-axis. Dot size represents nominal p-value. **d** Volcano plot of differentially expressed genes in 3D compared to 2D cultures. 869 genes are upregulated (red) and 1010 genes are downregulated (blue) (adjusted p-value (padj) < 0.05, log2FC > 1), not significantly deregulated genes are indicated in grey. **e** Scatter plot of GSEA analysis showing the 15 top and bottom enriched GO biological processes in 3D cultures compared to 2D cultures. Nominal enrichment score (NES) is represented on the x-axis. Dot size represents the gene ratio and colour represents nominal p-value

To characterise the differences between our primary 2D monolayer cultures and the newly established spheroid model and how these differences related to the corresponding parental tumour tissues, we compared the transcriptomes of 13 samples using 3 conditions. Principal component analysis showed that samples from each condition (2D, 3D, Tissue) predominantly grouped together in principal component space, indicating that transcriptomes derived from each condition showed low gene expression variance. Thus, transcriptome signatures are dominantly influenced by culture conditions instead of patient-specific characteristics. In addition, both cell culture clusters showed low variance between each other, but similar variance compared to tissue (Fig. 5a). These findings were confirmed by hierarchical clustering analysis which revealed the same pattern, demonstrating a cluster of tissue samples and a cluster of cell culture samples, which was further divided into two clusters separating monolayers (2D) and spheroids (3D) (Fig. 5b). GSEA analysis revealed that tissue clusters were mainly enriched for processes associated with glucose import and the catabolism of biomolecules (Fig. 5c). Details of all enriched terms can be found in Additional file 2.

Elucidating the differences between the two cell culture models (2D and 3D) allowed us to identify the distinct transcriptome signatures between monolayer cultures and spheroid cultures. Differential expression analysis revealed 1879 significantly deregulated genes, of which 869 genes were upregulated and 1010 were downregulated in 3D compared to 2D (Fig. 5d). Additional file 3 shows an overview of all deregulated genes. GSEA analysis demonstrated enriched processes including GO terms associated with histone demethylation, regulation of extracellular matrix organization, regulation of epithelial-to-mesenchymal transition (EMT) and the Notch signalling pathway in 3D cultures (Fig. 5e). The top 15 enriched biological processes in 2D compared to 3D included terms associated with mitochondrial structures and branched-chain amino acid metabolism.

### Meningioma spheroids show an enhanced expression of genes related to EMT in spheroid cultures compared to monolayer cultures

**Fig. 6:**
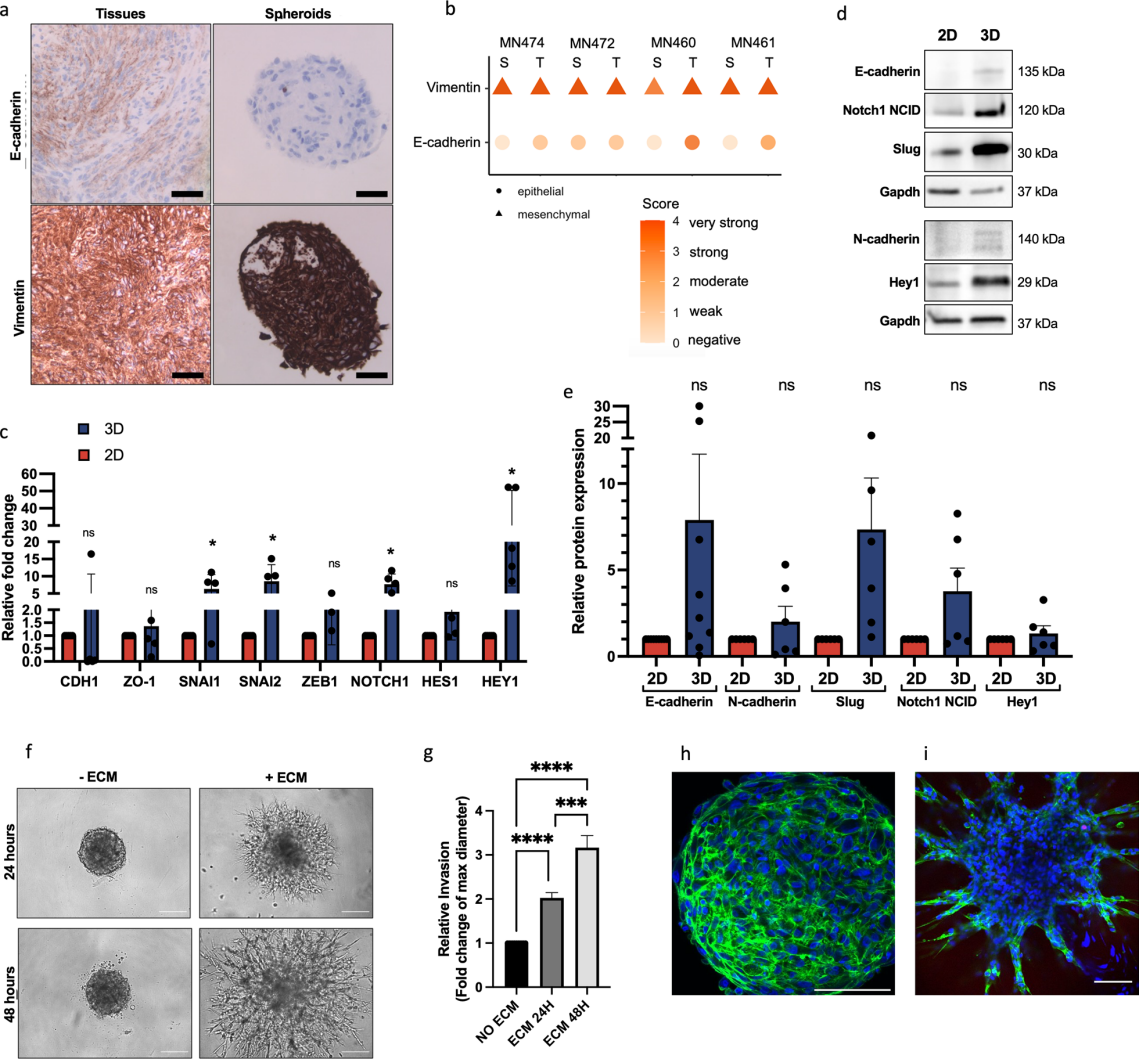
Enhanced expression of genes related to EMT in spheroid cultures compared to monolayer cultures. **a** Representative immunostaining images of patient-derived meningioma spheroids and matched tumour tissue (n = 4) stained for anti-E-cadherin, and anti-vimentin (Leica IM8). Scale bars: 200 µm. **b** Plot of immunoscores of immunostaining of E-cadherin (epithelial indicated as circle) and Vimentin (mesenchymal indicated as triangle) in spheroids (S) and matched tumour tissue (T) (n = 4). Colour represents immunoscore of 0–4 (0 = negative, 1 = weak, 2 = moderate, 3 = strong, 4 = very strong) **c** Relative gene expression of a panel of EMT markers: CDH1 (E-cadherin), ZO-1 (TJP1), Snai1, Snai2, Zeb1, Notch1, Hes1, and Hey1 in 2D monolayer cultures (red) compared to matched spheroids (3D) (blue) (n = 5). **d** Representative western blot and **e** quantification showing the expression E-cadherin, Notch1 NICD, Slug, N-cadherin, Hey1 in spheroids (3D) compared to patient matched monolayers (2D). Expression is shown as the relative increase compared to monolayers. **f** Representative phase-contrast microscopy images of WHO grade 2 spheroids with and without ECM (Matrigel) at 24 h and 48 h time points. Scale bars indicate 100 µm. **g** Bar graph showing the fold change of the max. diameter for spheroids embedded in ECM compared to spheroid controls that were not embedded. Max.diameter was measured using ImageJ. One-way ANOVA with Tukey’s multiple comparisons test was used for statistical evaluation. ***p < 0.001, ****p < 0.0001. **h** Fluorescence microscopy image showing F-actin (phalloidin, green) and nuclei (DAPI, blue) in spheroids without ECM and **i** with ECM showing invadopodia -like projections migrating into the ECM (Matrigel) at 48 h. Scale bars indicate 100 µm

In many cancers, EMT is associated with tumour progression, treatment resistance, invasion capacity and poor prognosis [25, 45]. We interrogated our transcriptomic data set for the expression of two genes associated with meningiomas and EMT: VIM encoding for vimentin (mesenchymal) and CDH1 encoding for E-cadherin (epithelial) [46–48]. The gene encoding vimentin was not significantly deregulated in our transcriptomic dataset, the CDH1 gene encoding for E-cadherin was significantly downregulated in 3D compared to 2D (log fold change: − 3.01, padj < 0.0001) as well as significantly downregulated in 2D compared to tissue (log fold change: − 1.94, padj < 0.02) and in 3D compared to tissue (log fold change: − 4.95, padj < 0.0001). To confirm enrichment of EMT in spheroid cultures on protein level we performed immunostaining for vimentin and E-cadherin in our spheroid cultures and matched patient tissues. Indeed, vimentin was strongly expressed in meningioma spheroids and tissues (average immuno scores of 4, and 3.75 for tissues and spheroids respectively) while expression of the epithelial marker E-cadherin was weak in spheroids and tissues (average immuno scores of 1.75, and 0.25 for tissues and spheroids respectively) (Fig. 6a, b). Next, to test the hypothesis that the spheroid model is a better model for EMT, we focused on the differences between the two in vitro models. Therefore, we investigated changes in the expression of markers associated with EMT in spheroids compared to monolayer cultures by western blotting and qPCR analysis. Since the Notch signalling pathway was also identified as enriched in spheroids and has been implicated to induce EMT, components of the Notch pathway, specifically Notch1, were also included in the analysis. Consistently, an increase in the gene expression of EMT transcription factors was observed (Fig. 6c). For Snai1 (encodes the Snail protein) a significant average 6.3-fold increase (*p* < 0.05) and for Snai2 (encodes the Slug protein) a significant average 8.5-fold increase was detected (*p* < 0.05) in spheroid cultures compared to matched monolayer cultures. For Notch1, an average 7.7-fold increase (*p* < 0.01) in RNA expression was observed with the downstream effectors Hes1 and Hey1 demonstrating a fold increase of 1.9 (*p* = 0.28) and 28.8 (*p* < 0.05) respectively (Fig. 6c). For the epithelial markers CDH1 (encoding for E-cadherin) and ZO-1 a change in gene expression was not observed (Fig. 6c) (CDH1 *p* = 0.51; ZO-1 *p* = 0.55). Interestingly, 4 out of 5 patient-derived spheroids showed an average decrease of 96% in E-cadherin expression, consistent with an increase of mesenchymal genes, while one sample showed a 16-fold increase. Similar to the RT-PCR a trend of increased protein expression of N-cadherin, Notch1 NICD, Hey1 and Slug was observed by western blotting although this increase was not significant due to the variability between patient samples (Fig. 6d, e). Similarly, a decrease in E-cadherin expression was only detected in some of the samples (Fig. 6d, e). These results suggest that predominantly mesenchymal markers are increased in spheroid cultures while epithelial markers remain similarly expressed.

### WHO grade 2 spheroids display invasion capacity when embedded in extracellular matrix (ECM)

The WHO classification includes brain invasion as a stand-alone criterium for WHO grade 2 meningiomas [49]. Moreover, an upregulated expression of the mesenchymaI proteins Snail and Slug has been shown in atypical grade 2 meningioma tissues compared to grade 1 tissues [50]. Considering the association between the mesenchymal phenotype and invasion, we analysed the functional invasiveness displayed by WHO grade 2 spheroids using a 3D Matrigel invasion assay. In agreement with the observation of an enhanced mesenchymal phenotype, embedded spheroids displayed observable protrusions in the Matrigel within 24 h, which was observed to significantly increase after 48 h (*p* < 0.001) (Fig. 6f, g). At 48 h, using F-actin and DAPI immunofluorescent staining, we observed a disorganization of the compact spheroids characterized by invadopodia-like projections migrating into the ECM away from the spheroid core in contrast to the spheroids that were not embedded in ECM, which retained their compact structure (Fig. 6h, i). Overall, these results demonstrate an enhanced mesenchymal expression signature in spheroids which indicates its functionality as in vitro cell culture tool to study EMT. Similar patterns of invasion were also observed for WHO grade 1 spheroids (Additional file 5: Fig. S2).

### Combined therapy of the MERTK/Flt3 inhibitor UNC2025 and HDAC inhibitor Trichostatin-A decreases spheroid viability and proliferation and reverses mesenchymal transition in meningioma spheroids

**Fig. 7:**
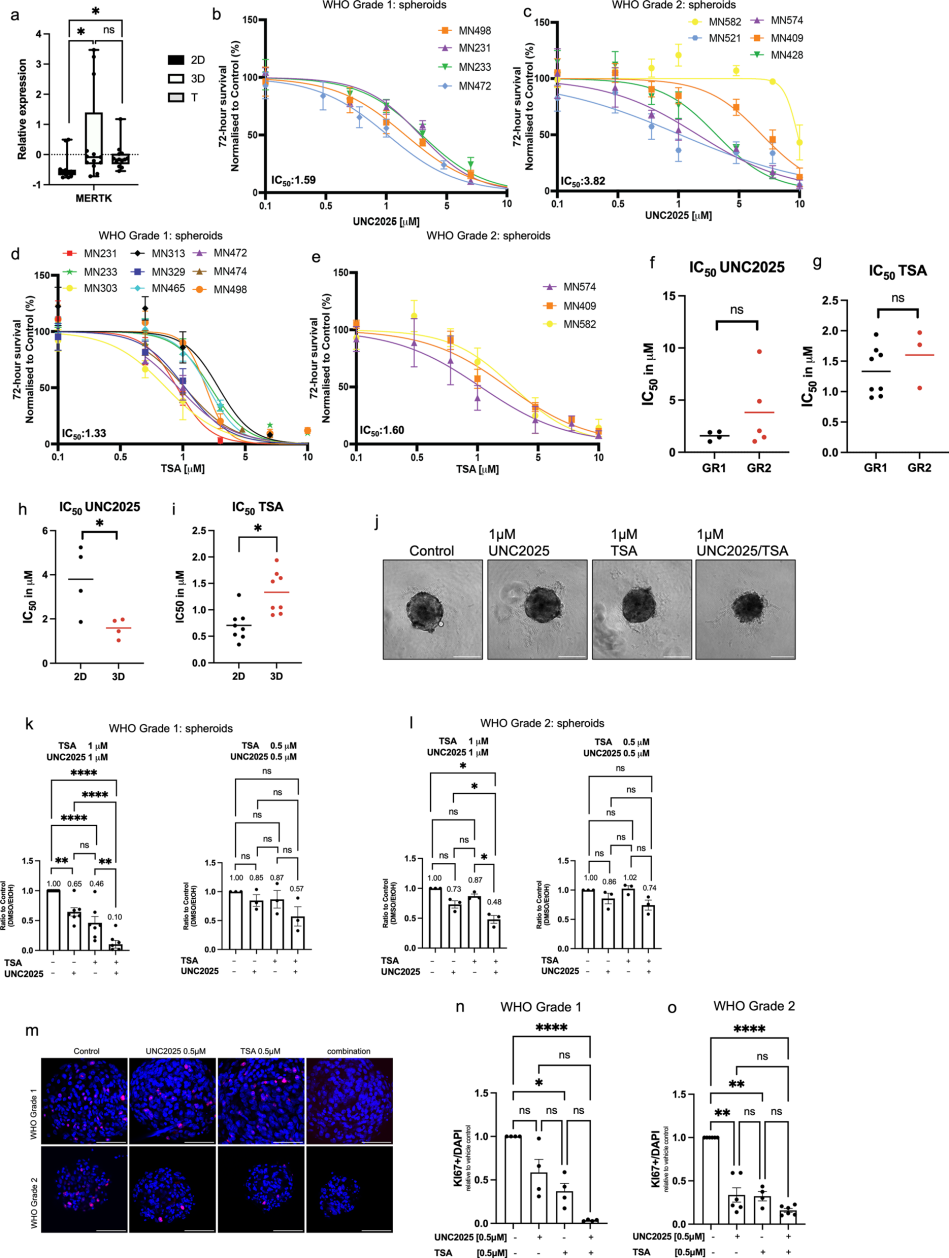
Combination therapy of UNC2025 and TSA decreases meningioma spheroid viability and proliferation. **a** Relative MERTK expression (RNAseq) in 2D, 3D and tissue. **b**-**e** Viability of **b** WHO grade 1 (n = 4) (average IC_50_ = 1.59 µM) and **c** WHO grade 2 spheroids following UNC2025 treatment (n = 5) (average IC_50_ = 3.82 µM) and **d** WHO grade 1 (n = 9) (average IC_50_ = 1.33 µM) and **e** WHO grade 2 (n = 3) (average IC_50_ = 1.60 µM) following TSA treatment at 72 h. Error bars indicate standard error of mean. **f**, **g** Average IC_50_ for **f** UNC2025 (*p* = 0.263) and **g** TSA (*p* = 0.371) in WHO grade 1 and grade 2 spheroids and for **h** UNC2025 (*p* < 0.05) and **i** TSA (*p* < 0.05) in grade 1 2D (black) and 3D (red) cultures. Patient matched samples were used. Student’s t-test; ns = not significant, *p < 0.05 **j** Representative bright field images of spheroids after monotherapy and combination therapy of UNC2025 and TSA at 1 µM UNC2025 and 1 µM TSA. Scale bar = 200 µm (Leica IM8). **k** Relative WHO grade 1 (n = 6) and **l** WHO grade 2 (n = 4) spheroid viability at 72 h treatment with 1 µM and 0.5 µM TSA and UNC2025 (n = 6). Each dot represents an individual sample. **m** Representative immunofluorescence images of Ki67 (red) in WHO grade 1 (top) (n = 4) and WHO grade 2 (bottom) (n = 6) spheroids following 72 h of mono or combination treatment with 0.5 µM TSA and 0.5 µM UNC2025. Cell nuclei are stained with DAPI (blue). Scale bar = 100 µm. (Leica confocal SP8. (n, o) Quantification of Ki67 positive cells relative to DAPI (nuclei) after combination treatment with 0.5 µM TSA and 0.5 µM UNC2025 in **n** WHO grade 1 (n = 4) and **o** WHO grade 2 (n = 6) spheroids. Data is represented as relative to vehicle-treated controls. ns = not significant, *p < 0.05, **p < 0.01, ****p < 0.0001. One-way ANOVA with Dunett’s test for multiple comparisons

The TAM receptor family of tyrosine kinases, MERTK, Axl and Tyro3, inhibition play a role in tumour development in several cancers and a growing body of evidence points towards a role for TAM receptor signalling in the initiation of EMT [29, 51, 52]. TAM receptor family expression has been previously associated with meningioma biology suggesting this receptor family as potential therapeutic target for meningiomas. Indeed, unpublished work by our group has demonstrated the upregulation of MERTK expression in meningiomas. Furthermore, we found MERTK expression was significantly increased in spheroid cultures compared to monolayer cultures in our transcriptomics dataset (Fig. 7a). Therefore, we chose to investigate the effect of the MERTK/Flt3 inhibitor UNC2025 on several aspects of EMT, and its effect on spheroid viability and proliferation. Furthermore, to enhance therapeutic efficacy, we sought to develop a combination strategy using the HDAC inhibitor Trichostatin A (TSA), which has been associated with the reversal of EMT in several cancers [53, 54] and has been suggested as novel therapeutic approach for meningiomas [55, 56].

Treatment of primary meningioma spheroids with increasing concentrations of UNC2025 effectively decreased spheroid viability at µM range, with an average IC50 of 1.76 µM for meningioma WHO grade 1 spheroids and 3.82 µM for meningioma WHO grade 2 spheroids (Fig. 7b, c). In addition, single-dose treatment with TSA effectively decreased meningioma spheroid viability with an average IC_50_ of 1.25 µM in meningioma WHO grade 1 and 1.60 µM in meningioma WHO grade 2 spheroids (Fig. 7d, e). For both therapies, WHO grade 2-derived spheroids showed a higher drug resistance compared to WHO grade 1 although this difference was not significant (Fig. 7f, g). In addition, matched monolayer cultures derived from the same patient samples displayed altered sensitivity towards both compounds compared to spheroids (Fig. 7h, i) demonstrating the significance of using appropriate in vitro models for drug validation studies. Importantly, combined treatment of meningioma spheroids showed a strong synergistic decrease in spheroid viability for both WHO grade 1 and 2 meningiomas with a dose of 1 µM UNC2025 and 1 µM TSA (Fig. 7j, k, l). Additionally, combined therapy with half of this dose (0.5 µM UNC2025 and 0.5 µM TSA) synergistically reduced spheroid proliferation demonstrated by a decrease in Ki67 positive cells, which serves as a marker for proliferation (Fig. 7l, m, n).

**Fig. 8:**
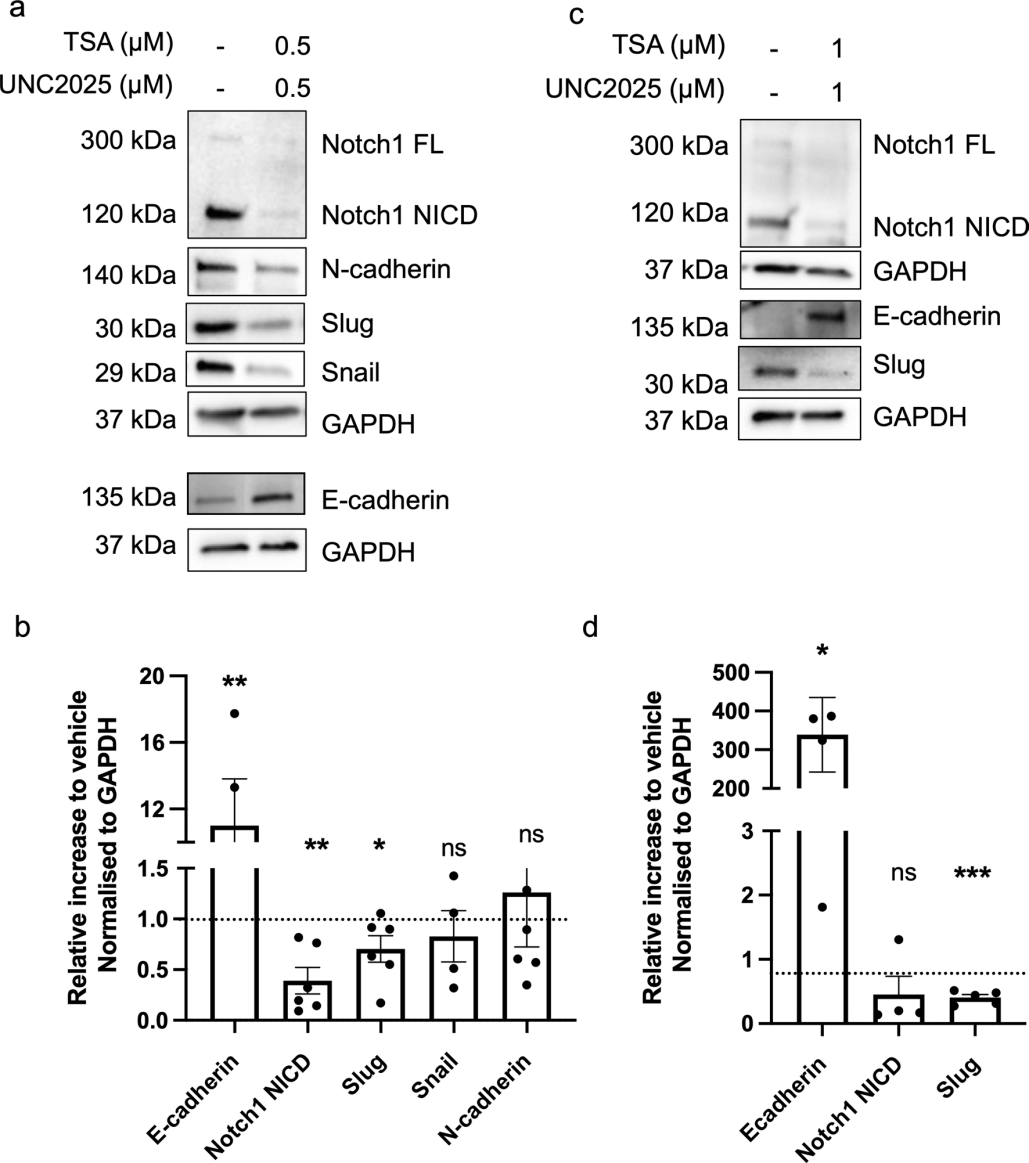
Combination therapy of UNC2025 and TSA decreases expression of meningioma EMT-related markers. **a** Representative western blot and **b** quantification showing E-cadherin, Notch1 (FL and NICD), Slug, Snail and N-cadherin expression in WHO grade 1 spheroids after combination therapy UNC2025 and TSA at a concentration of 0.5 µM. **c** Representative western blot and **d** quantification showing E-cadherin, Notch1 (FL and NICD) and slug expression in WHO grade 2 spheroids after combination therapy using UNC2025 and TSA at a concentration of 1 µM. GAPDH was the loading control. Paired t-test was used for statistical evaluation: *p < 0.05, **p < 0.01, ***p < 0.001, ns = not significant, FL = full length., NICD = Notch intracellular domain

**Fig. 9:**
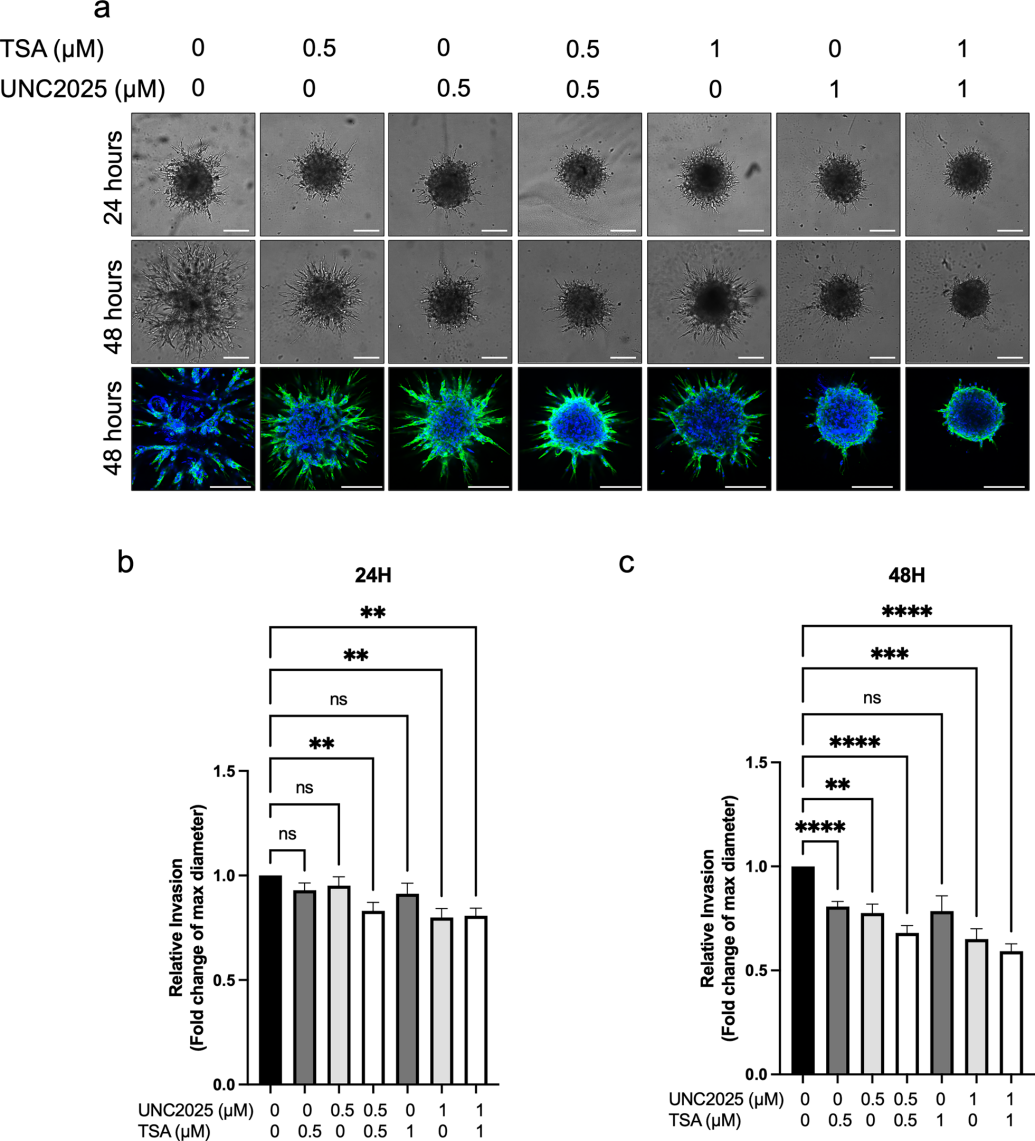
UNC2025 and TSA treatment abrogate invasion capacity of WHO grade 2 spheroids (**a**) Representative images of the 3D Matrigel invasion assay showing invasion capacity of WHO grade 2 spheroids at 24 (top panel) and 48 (middle, bottom) hours following monotherapy and combination therapy of UNC2025 and TSA at various concentrations (0.5 µM; 1 µM) compared to vehicle control (0.1% DMSO, 0.1% Ethanol) (n = 3). Images were taken using bright field microscopy (top and middle panel), and confocal microscopy (bottom panel). Scale bar in each panel represents 200 µm. Cell nuclei are stained with DAPI (blue) and Phalloidin (green). **b**, **c** Bar graphs showing the quantification of relative invasion at **b** 24 h and **c** 48 h presented as max. diameter of the total invasive zone in µm. Error bars indicate standard error of mean. One-way ANOVA with Dunett’s test for multiple comparisons was used for statistical evaluation; ns = not significant, **p < 0.01, ***p < 0.001, ****p < 0.001

We then investigated whether the combination therapy of UNC2025 and TSA had an effect on the expression of EMT-associated genes and proteins. Combination treatment of 72 h with 0.5 µM UNC2025 and 0.5 µM TSA resulted in a significant 11-fold increase in E-cadherin expression in WHO grade 1 spheroids (*p* < 0.01). Moreover, we detected a modest but significant decrease in the EMT-associated proteins Slug (1.4-fold decrease, *p* < 0.05) and the active intracellular domain of Notch1 (NICD) (2.6-fold decrease, *p* < 0.01) (Fig. 8a, b). For the mesenchymal protein Snail (1.2-fold decrease, *p* = 0.55), but not N-cadherin (1.26-fold increase, *p* = 0.65), a decreasing trend was observed. Similarly, WHO grade 2 derived spheroids treated with a higher dose of 1 µM UNC2025 and 1 µM TSA showed a significant 339-fold increase in E-cadherin and a significant 2.5-fold decrease in Slug (Fig. 8c, d). These results suggest that the combination strategy of UNC2025 and TSA is potent to induce E-cadherin to a strong level but only moderately reduces the expression of mesenchymal proteins in meningioma spheroids. Next, we tested if these changes in protein and gene expression were sufficient to have a functional effect, the spheroid invasive capacity of WHO grade 2 meningiomas after treatment. Indeed, combination therapy using a dose of 0.5 µM and 1 µM UNC2025 and TSA significantly decreased the spheroid matrigel invasion capacity after both time points compared to vehicle-treated controls (Fig. 9). In addition, monotherapy of UNC2025 at both concentrations and monotherapy with TSA at 0.5 µM also significantly decreased invasion after 48 h (Fig. 9c). Strikingly, although approaching significance (*p* = 0.058), TSA at the higher dose of 1 µM did not significantly decrease invasion. Interestingly, after 24 h, 1 µM UNC2025 decreased the invasion capacity to a similar level as the combination strategy at that same dose. Altogether, the overall effect of treatment with both UNC2025 and TSA and the combination of the two compounds showed an inhibitory effect on spheroid invasion capacity which is indicative of a functional effect on EMT.

## Discussion

Development of a good in vitro system to model the complexity of meningioma pathology is essential for investigating drug response and developing novel therapeutics. In this study, we established an easy-to-use patient-derived spheroid model of meningioma with high efficiency and fast result turn-around that maintained the morphological and molecular features of their parental tumours and serves as a model for EMT. Using this model, we demonstrated the therapeutic potential of the combination therapy of the MERTK inhibitor UNC2025 and the HDAC inhibitor Trichostatin A (TSA) to treat patient derived WHO grade 1 and grade 2 meningiomas. Several meningioma 3D culture models have been previously established, although none of these have yet been widely adopted [20, 57–59]. The method established here uses a scaffold-free approach, is easy to handle and highly reproducible. This makes it simple, less time-consuming, and inexpensive when compared to other 3D techniques, such as organoids [21].

The immune microenvironment of meningioma has been reported to influence tumour development and growth [18, 58]. Specifically, tumour-associated macrophages have been demonstrated to affect drug response and have even been shown to contribute to drug resistance in several cancers, including meningiomas [60, 61]. Therefore, we deliberately generated primary multicellular spheroids to preserve the intricacies of the immune microenvironment and the inherent multicellular organization of differentiated meningioma cells using conditions allowing differentiation. Characterisation of our novel spheroid model revealed the presence of a macrophage population, as evidenced by immunostaining for macrophage markers anti-CD68 and anti-CD163 [62, 63]. Furthermore, several disease-causing mutations, including NF2, TRAF7, KLF4, AKT1 and SMO, have been described in meningiomas and compounds specific for driver mutations are currently under investigation in clinical trials [64, 65]. This signifies the importance of maintaining the mutational landscape of meningiomas during in vitro experiments. Here, we showed that our spheroid cultures maintained the genomic alterations of parent tissues. Together, these results show that our model can effectively reflect components of the meningioma microenvironment and the genomic background of meningiomas, and thus is a robust tool to assess the efficiency of compounds targeting specific genomic alterations and immunotherapy.

We showed that our spheroid model displayed low levels of growth when assessing the growth dynamics based on increase in diameter (Fig. 2e). However, when assessing the percentage of cells positively stained for the proliferation marker Ki67, some spheroids displayed a high level of proliferation compared to tumour tissues[66]. This could possibly be due to an introduced bias by the method of counting, since one cannot count the entirety of the tumour. Meningiomas demonstrate highly variable levels of proliferation in different areas within the tumour, and taking an average from a number of areas could give an overall impression but does not adequately represent the variable areas. Therefore, assigning an immunoscore will give a more unbiased overview of the staining level and how this compared to the spheroid. The elevated levels of proliferation in the spheroids compared to the parental tumour tissues could be explained by the distance to nutrients in the medium. The average spheroid diameter after 6 days in culture is approximately 200–300 μm. Therefore, the maximum distance to the nutrients in the medium is approximately 150 μm, while in the tumour tissue, this distance to blood vessels can be larger, resulting in areas of less proliferation.

Although we used patient-derived materials, we observed significant differences between the transcriptome profiles of both cell culture models compared to matched patient tissues. These findings are not surprising as transcriptomic differences are to be expected due to the simplicity of in vitro modelling and loss of structures such as blood vessels and other cell types that are present in vivo. In our experiment, the transcripts that are detected from structures in the tissue that are not present in the cell cultures (e.g. blood vessels), cannot be separated from the transcripts that are directly derived from the tumour cells, which presents a limitation of our current study. To exclude the transcriptomic changes that are driven by the absence of these structures from the analysis, single-cell RNA sequencing should be carried out, which allows for comparing gene expression of exclusive populations such as meningioma cells in tissues to those in the in vitro cultures. Such analysis would give a broader overview of transcriptomic changes for different cell types in the tissues and in vitro models. However, comparison of the two in vitro models is not limited by this feature. Interestingly, GSEA analysis of the differentially expressed genes (DEGs) between our newly established spheroids and traditional monolayer cultures revealed an upregulation in genes associated with EMT, which is known to be relevant in meningioma progression [27, 47].

To confirm our findings, we showed that our spheroids expressed low levels of the epithelial protein E-cadherin (Fig. 6a), while showing high expression levels of the mesenchymal protein vimentin. Additionally, the expression of a panel of EMT-associated markers was analysed by qPCR and western blotting and revealed upregulation of the EMT-transcription factor genes snai1 and snai2 (encoding Slug), consistent with progression to EMT [28, 47]. While we observed a relative increase of all mesenchymal proteins in our western blotting panel, these did not reach significance. This is likely due to patient variability, which resulted in a high standard error of the results. Furthermore, with qPCR we could confirm a decrease in CDH1 (encoding E-cadherin) expression corresponding with EMT in 4 out of 5 patient samples, as well as in the transcriptomic dataset, although western blotting showed an increase in E-cadherin expression in some and decreased expression in other patient samples. Although an increase in E-cadherin is in contrast with EMT progression, this increase is likely due to the role of E-cadherin in cell–cell adhesion, which is increased when cells are grown in 3D compared to 2D [66, 67]. Besides canonical EMT proteins, we could also confirm an increased expression of Notch1 signalling proteins, Notch1, Hes1 and Hey1, confirming the GSEA enrichment analysis. Together, these results suggest that meningioma spheroids are indeed progressing towards an increased mesenchymal state but have not fully completed EMT [45]. The phenomenon of cells acquiring EMT characteristics in spheroid cultures is not unique to our model. Similar findings were reported by several others [68–73]. For instance, Wong and colleagues comprehensively characterized the transcriptomes of placental extravillous trophoblast spheroids and found significant up-regulations in genes and proteins related to EMT, cell–cell contact, angiogenesis and invasion/migration as compared to monolayer cultures [69]. Similarly, Kuo et al*.* demonstrated that 3D spheroid culture of human epithelial ovarian cancer cells using microfluidic chips resulted in the acquisition of mesenchymal traits, as evidenced by an increased expression of the mesenchymal proteins N-cadherin, vimentin and fibronectin and a concomitant decrease in expression of CD326, an epithelial cell adhesion molecule, in comparison to traditional monolayer cultures [71]. One of the suggested mechanisms behind this phenomenon is the microenvironment of the spheroids [69, 72]. For instance, oxygen gradients caused by limited oxygen diffusion result in hypoxic conditions in spheroid cores, which has been shown to result in hypoxia-induced EMT [72]. Furthermore, the mitogenic growth factors EGF and FGF, commonly supplemented as components of spheroid culture media; including GFS, have been shown to trigger EMT [72]. Indeed, the maintenance of primary meningioma cells under serum-free conditions supplemented with these factors was shown to spheroid cultures enriched for the stem-like cell population. These meningioma stem-like cells are characterized by mesenchymal phenotypes [74, 75]. Therefore, the presence of EGF and FGF in our culture medium could indicate presence of stem-like cells within these cultures, which may have influenced the observed EMT. In addition, the presence of immune cells has been shown to induce EMT. For instance, exosomes secreted by M2-macrophage were shown to activate TGFβ-signalling mediated EMT in meningioma cells, which enhanced their migratory and invasive ability [76]. In addition to expression of EMT markers, we showed that our meningioma spheroids can effectively mimic invasion, a process that has been associated with cells undergoing EMT. Due to the characteristics of this assay, interference of proliferation could not be completely ruled out. However, this protocol has been broadly accepted as 3D invasion assay in literature [52, 77, 78]. To our knowledge, this model is the first 3D model for meningiomas that can mimic invasiveness, which cannot be treated by surgery and thus requires drug treatment [74, 76]. Altogether, this shows that our model not only reflects essential features of meningioma tissues such as the meningioma immune microenvironment, genomic alterations and histology but also allows to investigate EMT and invasiveness.

We then used our spheroid model and provided evidence for the potential of the MERTK inhibitor UNC2025 and the HDAC inhibitor TSA as a novel therapeutic strategy for the treatment of meningiomas. Importantly, similar inhibitors targeting HDAC and MERTK are currently in ongoing clinical trials [75, 77]. We showed that treatment with single doses of UNC2025 and TSA was effective to inhibit spheroid viability and proliferation for spheroids derived from WHO grade 1 and 2 meningiomas. Since the proliferation levels of these tumours are low, the outcome of the proliferation assay should be interpreted carefully and assessing the change in viability of the spheroid pre- and post-treatment is a more robust test to predict therapy response. Furthermore, it must be noted that WHO grade 2 cells are isolated using higher levels of FBS compared to the isolation of grade 1 cells, which could have contributed to the difference in effect between the two WHO grades, although for spheroid culture the same medium was used [79–82]. More importantly, we observed a differential drug response of monolayers treated with a single dose of either UNC2025 or TSA compared to spheroid response, although a decrease in viability and proliferation was observed in both models. We noticed spheroids had an increased sensitivity towards monotherapy of UNC2025 and a decreased sensitivity towards monotherapy of TSA compared to monolayers, signifying the importance of 3D cell culture in drug development studies. These results are in agreement with previous reports in the literature that demonstrated spheroids have decreased drug sensitivity compared to monolayer cultures [78, 83–85], although, enhanced drug sensitivity in spheroids has also been reported, suggesting that drug sensitivity is culture system and cell type dependent [86]. It is likely that this difference in UNC2025 sensitivity of meningioma patient-derived spheroids and monolayers is caused by the decreased gene expression of the target MERTK in monolayers (Fig. 7a), making the cells less dependent on MERTK signalling. This underlines the importance of using the right models to predict the right tumour response. Moreover, in addition to meningioma cells, MERTK is expressed by tumour-associated macrophages [51, 79]. We demonstrated the presence of macrophages in our spheroid cultures. Hence, the altered macrophage-tumour cell interaction in spheroids could potentially affect their crosstalk, which in turn, could influence spheroid sensitivity to these drugs.

Furthermore, we showed that combined treatment with UNC2025 and TSA synergistically inhibited the viability and proliferation of meningioma spheroids, which might allow for the administration of lower drug concentrations in patients, reduced off-target effects and improved overall clinical outcomes [87]. Furthermore, we provided evidence for the potency of this combination therapy to induce E-cadherin expression alongside the repression of the mesenchymal proteins Slug and Notch1 NICD. However, combination therapy did not decrease N-cadherin expression or Snail expression, which suggests that only a partial reversal of EMT is achieved [88]. It is commonly accepted that loss of E-cadherin can initiate cell migration and invasion, due to loss of E-cadherin mediated cell–cell adhesion [89]. Therefore, treatment that leads to re-expression of E-cadherin could be an attractive strategy to decrease brain invasion. Indeed, our results showed evidence that re-expression of E-cadherin in meningiomas, induced by combination therapy is sufficient to decrease spheroid invasive capacity, despite unchanged levels of mesenchymal N-cadherin [80–82, 86, 90–96].

## Conclusions

In conclusion, we established a novel patient-derived meningioma spheroid model that resembled morphology, molecular features, and immune microenvironment of meningioma parent tissues. With the enhanced EMT gene expression profile and invasive capacity of these spheroids compared to monolayers, we propose that our model can be used as drug screening tool to assess the efficacy of drug compounds targeting EMT of meningiomas. Finally, we identified combination therapy of UNC2025 and TSA as a potential systemic therapy modality for treatment of WHO grade 1 and grade 2 meningiomas. We believe that implementing this model for future drug development experiments will improve accuracy and can ultimately result in decreased failure rates of clinical trials.

### Supplementary Information

**Additional file 1.** All driver mutations in matched spheroids and tissues.

**Additional file 2.** Enrichment data of GSEA analyses.

**Additional file 3.** List of significantly deregulated genes (DEG) between 3D and 2D, 3D and Tissue and 2D and Tissue.

**Additional file 4:** **Fig. S1.** Spheroids can be successfully formed from higher passages. Representative phase-contrast microscopy images of WHO grade 1 spheroids (MN708, MN611, MN656) (n=3) and WHO grade 2 spheroids (MN660) 3-days post seeding derived from attached cells at P1, P2 and P3. Scale bars indicate 100μm.

**Additional file 5:** **Fig. S2.** WHO grade 1 spheroids display invasion into ECM Representative phase-contrast microscopy images of WHO grade 1 spheroids (MN525, MN595) embedded in ECM (Matrigel) at 48h time points (n=2). Scale bars indicate 200μm.

## Data Availability

All data generated or analysed during this study are included in this published article and its additional files.

## Abbreviations

2D: Two-dimensional
3D: Three-dimensional
DEG: Differentially expressed genes
EMT: Epithelial-to-mesenchymal transition
FDR: False-discovery rate
FPKM: Fragments per kilobase million
GFS: Growth factor supplemented medium
GO: Gene ontology
GSEA: Gene set enrichment analysis
H&E: Hematoxylin and Eosin
HDAC: Histone deacetylase
MERTK: Mer tyrosine kinase
NICD: Notch intracellular domain
NES: Nominal enrichment score
SSTR2: Somatostatin receptor 2
TSA: Trichostatin A
ULA: Ultra low adherence
WHO: World health organisation
