# Supplementary figures and images for: A novel patient-derived meningioma spheroid model as a tool to study and treat epithelial-to-mesenchymal transition (EMT) in meningiomas
Source: Acta Neuropathol Commun. 2023 Dec 15;11:198. doi: 10.1186/s40478-023-01677-9 (PMC10725030; doi:10.1186/s40478-023-01677-9)

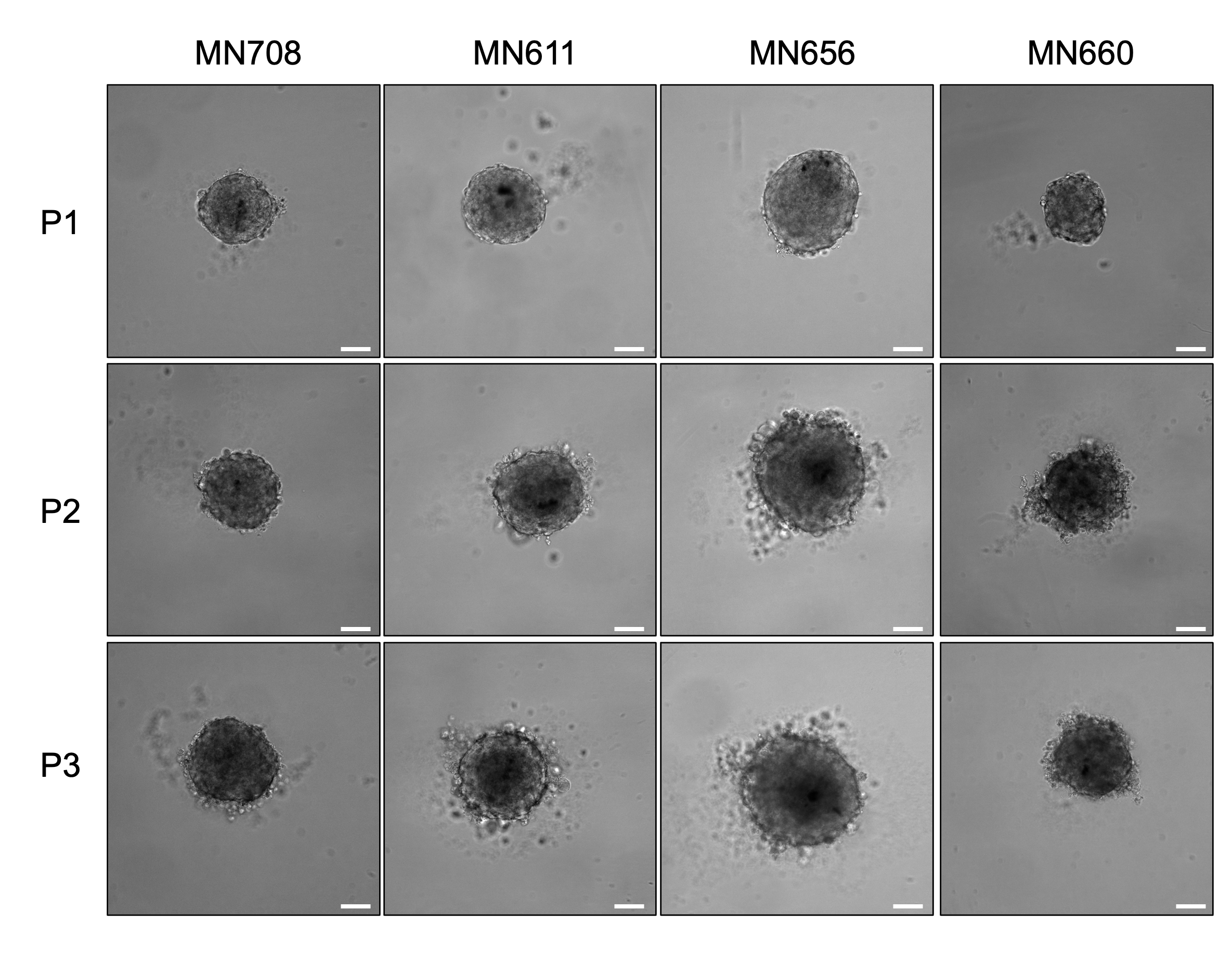

Supplement: Supplementary file 4 — Additional file 4: Fig. S1. Spheroids can be successfully formed from higher passages. Representative phase-contrast microscopy images of WHO grade 1 spheroids (MN708, MN611, MN656) (n=3) and WHO grade 2 spheroids (MN660) 3-days post seeding derived from attached cells at P1, P2 and P3. Scale bars indicate 100μm. [file 40478_2023_1677_MOESM4_ESM.png]

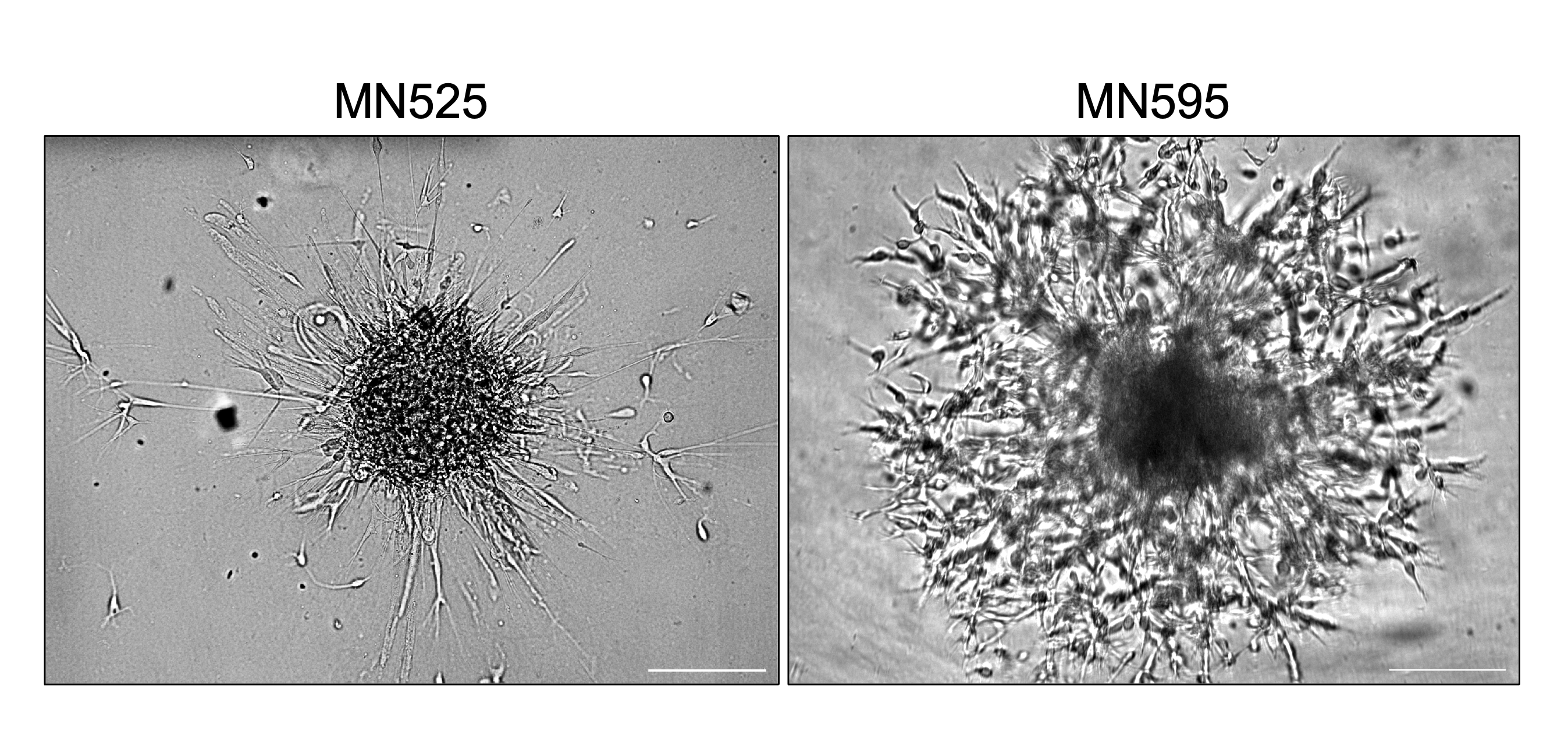

Supplement: Supplementary file 5 — Additional file 5: Fig. S2. WHO grade 1 spheroids display invasion into ECM Representative phase-contrast microscopy images of WHO grade 1 spheroids (MN525, MN595) embedded in ECM (Matrigel) at 48h time points (n=2). Scale bars indicate 200μm. [file 40478_2023_1677_MOESM5_ESM.png]
